# Immunogenicity of a Dendrimer B_2_T Peptide Harboring a T-Cell Epitope From FMDV Non-structural Protein 3D

**DOI:** 10.3389/fvets.2020.00498

**Published:** 2020-08-11

**Authors:** Rodrigo Cañas-Arranz, Patricia de León, Mar Forner, Sira Defaus, María J. Bustos, Elisa Torres, David Andreu, Esther Blanco, Francisco Sobrino

**Affiliations:** ^1^Centro de Biología Molecular “Severo Ochoa” (CSIC-UAM), Madrid, Spain; ^2^Departament de Ciències Experimentals i de la Salut, Universitat Pompeu Fabra, Barcelona, Spain; ^3^Centro de Investigación en Sanidad Animal (CISA-INIA), Madrid, Spain

**Keywords:** FMDV, vaccines, dendrimer peptides, T-cell epitopes, swine

## Abstract

Synthetic dendrimer peptides are a promising strategy to develop new FMD vaccines. A dendrimer peptide, termed B_2_T-3A, which harbors two copies of the major FMDV antigenic B-cell site [VP1 (140–158)], covalently linked to a heterotypic T-cell from the non-structural protein 3A [3A (21–35)], has been shown to protect pigs against viral challenge. Interestingly, the modular design of this dendrimer peptide allows modifications aimed at improving its immunogenicity, such as the replacement of the T-cell epitope moiety. Here, we report that a dendrimer peptide, B_2_T-3D, harboring a T-cell epitope from FMDV 3D protein [3D (56–70)], when inoculated in pigs, elicited consistent levels of neutralizing antibodies and high frequencies of IFN-γ-producing cells upon *in vitro* recall with the homologous dendrimers, both responses being similar to those evoked by B_2_T-3A. Lymphocytes from B_2_T-3A-immunized pigs were *in vitro-*stimulated by T-3A peptide and to a lesser extent by B-peptide, while those from B_2_T-3D- immunized animals preferentially recognized the T-3D peptide, suggesting that this epitope is a potent inducer of IFN-γ producing-cells. These results extend the repertoire of T-cell epitopes efficiently recognized by swine lymphocytes and open the possibility of using T-3D to enhance the immunogenicity and the protection conferred by B_2_T-dendrimers.

## Introduction

Foot-and-mouth disease (FMD) is a highly contagious disease affecting cloven-hoofed animals that is caused by a virus belonging to the *Picornaviridae* family: FMD virus (FMDV). Although the mortality rate is low, FMD is feared in farm industry and animal health because, in an outbreak, massive culling of infected or suspected animals is mandatory, with devastating economic impact. In addition, FMD control is costly in endemic countries in which current vaccines based on inactivated viruses are being used for disease control (1). Nevertheless, several drawbacks associated with these vaccines have led FMD-free countries to follow non-vaccination policies, increasing the risk of disease reintroduction and severe outbreaks (2). Therefore, the development of safer and effective vaccines is a major priority for FMD control including those based on viral subunits (3–5). Targeting capsid protein VP1 for the induction of neutralizing anti-FMDV antibodies was one of the first attempts to produce peptide-based subunit vaccines. Among the advantages of peptide vaccines are: (i) safety, as non-infectious material is used, and no reversion to virulence is possible, (ii) DIVA condition (efficient serological distinction between infected and vaccinated animals), (iii) easy to handle and store (no cold chain is required), (iv) chemical stability, and (v) affordable large scale production. The first attempts to produce vaccines based on synthetic VP1 capsid protein were reported in the early 80s (6), but later reports evidenced the low immunogenicity of VP1, probably due to non-native folding when expressed in a non-capsid protein context. Since then, peptides corresponding to the G-H loop in VP1 have been used as the main component of FMD peptide vaccines (7–9). An important advantage of this B-cell epitope is that it is structurally continuous and easy to mimic as a peptide.

Despite the vaccine potential of FMDV peptides, the main limitation faced during decades was their weak immunogenicity when compared with conventional vaccines that use inactivated virus as immunogen (10), a limitation that may lead to selection of antigenic variants in partially immunized animals (11). Optimization of the B-cell sites and inclusion in peptide vaccines of specific T-cell epitopes recognized by different MHC molecules capable of evoking adequate T-cell responses, are requirements for optimal production of FMDV neutralizing antibodies (nAbs) and have therefore been included in the composition of linear vaccine peptides (12–15). Nevertheless, since classical linear peptides barely achieved levels of protection in livestock as those required for their use as commercial vaccines (11, 16), multimerization strategies have been developed to overcome this low-immunogenicity. One of these approaches relies on so-called multiple antigenic peptides (MAPs), in which the B-cell epitope branches out from a lysine core scaffold giving rise to a dendrimer display (17). Interestingly, two doses (2 mg each) of a dendrimer peptide displaying four copies of the G-H loop from a type C FMDV linked to a heterotypic and highly conserved T-cell epitope from FMDV 3A protein [3A (21–35)], were able to protect pigs against homologous FMDV challenge (18). More remarkably, downsized versions bearing two copies of the B-cell epitope afforded full protection in swine against an epidemiologically relevant type O FMDV even upon a single peptide dose (19, 20).

The protective responses elicited by B_2_T-3A and other related dendrimeric constructs (hereafter B_2_T-dendrimers), associate with the induction of high titers of nAb and the activation of specific lymphocytes that would provide T-cell help for effective production of nAbs (18, 19). Besides, such T-cell epitopes can also stimulate T-cell subsets leading to the expression of IFN-γ, a cytokine with a relevant role in the antiviral response (21). Thus, a further characterization of the functional role of the T-cell epitope(s) recognized by swine lymphocytes in the B_2_T-dendrimers is relevant to understand how they work and to design vaccine improvements. Moreover, the MHC restriction phenomenon can limit the recognition by T-cells of B_2_T-3A and related peptide dendrimers among different pig individuals as well as between FMDV host species (22, 23). This makes the functional characterization of B_2_T-dendrimers encompassing T-cell epitopes other than T-3A an interesting goal.

Here, we show that a B_2_T-dendrimer (termed B_2_T-3D) including the porcine T-cell epitope identified in the 3D FMDV protein [3D (56–70)] previously shown to be promiscuous and heterotypic T-cell epitope (24) can elicit in pigs nAbs titers and IFN-γ-producing cells at levels similar to those induced by the dendrimer peptide B_2_T-3A.

## Materials and Methods

### Peptides

The B-cell epitope from FMDV (O/UK/11/2001), VP1 (residues 140–158), and the T-cell epitopes 3A (residues 21–35) and 3D (residues 56–70) were synthesized by Fmoc-solid phase synthesis (SPPS), purified by reverse-phase liquid chromatography (RP-HPLC) and characterized by mass spectrometry (MS). B_2_T-dendrimers were prepared by conjugation in solution of two B-cell peptides containing and additional C-terminal Cys (free thiol form) with one T-cell epitope N-terminally elongated with two Lys residues followed by an extra Lys branching point further derivatized into two maleimide groups (Table 1). The B_2_T-3A and B_2_T-3D constructs were obtained via thiol–maleimide ligation at pH 6.0, purified by RP-HPLC and characterized by MS (18, 19, 25).

**Table 1:** B_2_T bivalent dendrimeric constructions.

**B**_****2****_**T dendrimers**		
**General structure**^**a**^	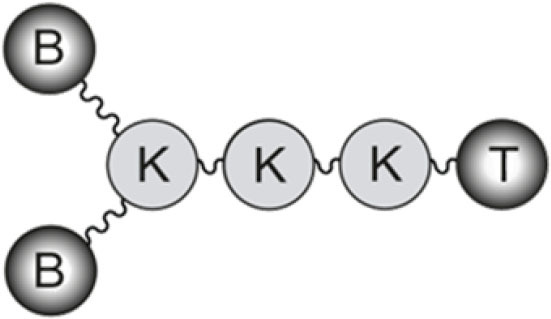	
**B epitope**	acetyl-PVTNVRGDLQVLAQKAARTC-amide	
**T-3A**	AAIEFFEGMVHDSIK-amide	
**T-3D**	IFSKHRGDTKMSAED-amide	
**Peptides**	**B**_**2**_**T-3A**	**B**_**2**_**T-3D**
**MW**^**b**^	6742.8 Da	6770.8 Da
**HPLC**^**c**^	6.9 min (98%)	5.1 min (98%)

a *B_2_T(mal) construct with the B epitope linked to a 3-maleimidopropionic acid unit attached in the Lys core and linked to the T cell epitope (T3-A or T-3D)*.

b *Experimental peptide mass obtained by LC/MS*.

c *Retention time on a C18 column (Luna, 4.6 × 50 mm, 3 mm; Phenomenex) eluted with a 20–60% linear gradient of solvent B (0.036% TFA in MeCN) into solvent A (0.045% TFA in H_2_O) over 15 min. In parenthesis, homogeneity of purified material*.

### Viruses

The FMDV stocks (O/UK/11/2001), O/SKR, O_1_Manisa, O_1_BFS (The Pirbright Institute, UK) and O_1_Campos (OPS-PanAftosa) were amplified in IBRS-2 cells and type C CS8-c1 virus (26) was amplified in BHK-21 cells.

### Animals

#### Mice

Groups of five 5-to-6-week-old outbred female mice were (Swiss ICR-CD1, Envigo) were maintained under standard housing conditions at CBMSO animal facility. Mice were immunized subcutaneously at days 0 and 21 with 100 μg of each B_2_T-dendrimer peptide emulsified in Montanide ISA 50V2 (Seppic-France) and euthanized at day 40. Blood samples were collected at days 0, 21, and 40 post-immunization (pi). Experimental procedures were conducted in accordance with protocols approved by the CSIC Committees on Ethical and Animal Welfare and by the National Committee on Ethics and Animal Welfare (PROEX 034/15).

#### Pigs

White cross-bred Landrace female pigs, 9–12 weeks-old (20 Kg), were maintained in a conventional farm facility at the Departamento de Reproducción Animal, INIA, Madrid. Groups of four pigs were immunized with B_2_T-3A or B_2_T-3D at day 0 with 2 ml of Montanide ISA 50V2 emulsion containing 2 mg of the corresponding peptide and boosted at day 21 pi. Two additional pigs were PBS-inoculated and maintained as controls. Blood samples were collected at days 0, 7, 14, 21, 28, 35, and 70 pi to obtain serum and peripheral blood mononuclear cells (PBMCs). The study was approved (CBS2014/015 and CEEA2014/018) by the INIA Committees on Ethics of Animal Experiments and Biosafety, and by the National Committee on Ethics and Animal Welfare (PROEX 218/14).

### Virus Neutralization Test (VNT)

Neutralization assays were performed in 96-well culture plates. Serial 2-fold dilutions of each serum sample (in DMEM containing 2% fetal bovine serum) were incubated with 100 infection units−50% tissue culture infective doses (TCID_50_)–of FMDV (O/UK/11/2001) for 1 h at 37°C. Then, a cell suspension of IBRS-2 cells in DMEM was added and plates were incubated for 72 h. Monolayers were controlled for development of cytopathic effect (cpe), fixed, and stained. End-point titers were calculated as the reciprocal of the final serum dilution that neutralized 100 TCID_50_ of homologous FMDV in 50% of the wells (19). For cross neutralization assays, incubation of sera with the panel of FMD viruses (O/SKR, O_1_Manisa, O_1_BFS, and O_1_Campos) that belonged to different type O topotypes was performed in parallel to that of the homologous isolate O/UK/11/2001 and the negative control type C CS8-c1 virus. The antigenic relationship of viruses was calculated by the ratio *r*_1_ = nAb titers against the heterologous virus/nAb titer against homologous virus, as reported (27).

### Detection of Anti-FMDV Antibodies by ELISA

Specific antibodies were assayed by ELISA as described (19) using plates coated with peptide B (1 μg) that were incubated with 3-fold dilutions of serum and detected using HRP-conjugated protein A. Plates were read at 450 nm and titers expressed as the reciprocal of the last serum dilution given an absorbance range of two standard deviations above the background (serum at day 0) plus 2 SD.

### PBMC Isolation and IFN-γ Detection by ELISPOT

Porcine PBMCs were isolated from blood samples collected in Vacutainer tubes EDTA-K2, diluted 1:1 in PBS and then used to obtain PBMC by density-gradient centrifugation with Histopaque 1077 (Sigma) and Leucosep tubes (Greiner Bio-One) as described (28). For the IFN-γ ELISPOT assay 2.5 × 10^5^ PBMCs were shed in triplicate wells of Immobilon-P plates (Merck Millipore) coated as reported (19) and *in vitro* stimulated with 50 μg/ml of their respective immunogenic peptides. As positive or negative controls, cells were incubated with 10 μg/ml of phytohaemagglutinin (Sigma) or only with medium, respectively. After 48 h at 37°C and 5% CO_2_, plates were washed and incubated with a biotinylated mouse anti-pig IFN-γ (clone P2C11, BD) followed by streptavidin:HRP (BD). The frequency of peptide-specific T-cells was expressed as the mean number of spot-forming cells/10^6^ PBMCs, with background values (number of spots in negative control wells) subtracted from the respective counts of stimulated cells. These experiments were performed using outbred domestic pigs with different individual genetic backgrounds. In any case, the levels of animal-to-animal variation did not exceed those observed in other related studies (11).

### Statistical Analyses

Differences among peptide-immunized groups in FMDV-antibody titers and number of IFN-γ producing cells were analyzed using the Student's *t*-test. Values are cited in the text as mean ± SD. All *p*-values are two sided, and *p* < 0.05 were considered significant. Statistical analyses were conducted using GraphPad Prism Software 5.0.

## Results

### Analysis of the Humoral Immune Response Elicited by B_2_T-3D FMDV Dendrimer in a Mouse Model

The mouse strain Swiss ICR (CD1®) offers the possibility of conducting immunogenic studies in outbred populations that mimic the heterogeneous genetic background of natural FMDV hosts (29). As previous results showed that peptide B_2_T-3A was able to induce significant levels of nAbs in outbred Swiss ICR mice, this strain was used to evaluate the immunogenicity of the dendrimer B_2_T-3D. To this end, groups of five mice were immunized with each B_2_T-dendrimer construction.

**Figure 1:**
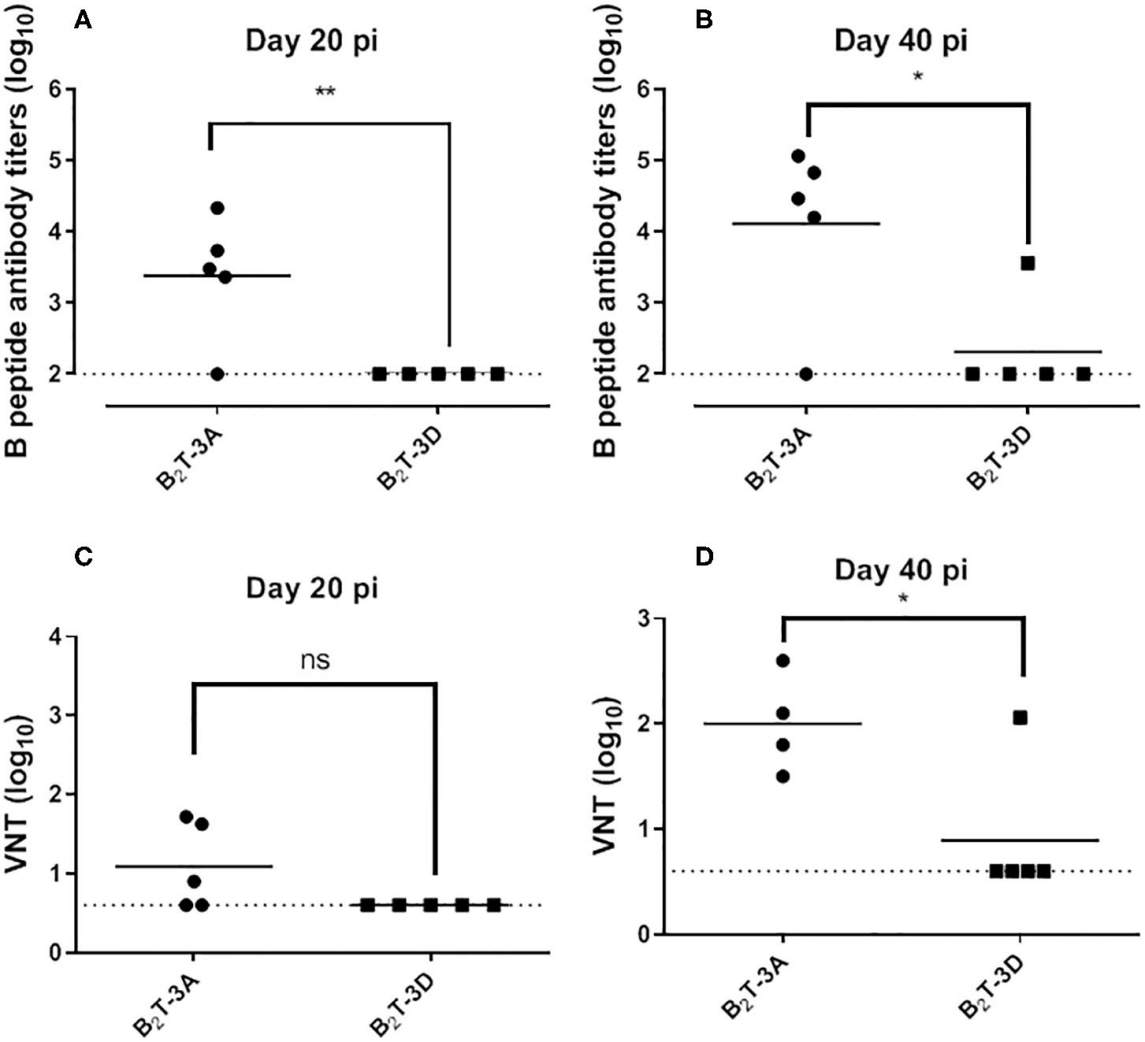
FMDV antibodies elicited in mice by B_2_T-3A and B_2_T-3D dendrimers. Total IgG antibodies against peptide B detected by ELISA in sera from immunized mice after the first (day 21 pi) **(A)** and the second peptide dose (day 40 pi) **(B)**. Virus neutralization titers, expressed as the reciprocal log_10_ of the last serum dilution that neutralized 100 TCID50 of homologous FMDV, after the first **(C)** and second peptide dose **(D)**. Each point represents the mean of a triplicate value of a single animal. Horizontal bars indicate the mean of each group. Statistically significant differences are indicated by asterisks (*) for *p* < 0.05 and (**) for *p* < 0.005; (ns) statistically non-significant difference. A representative experiment out of five is presented.

Total IgG antibodies against B-cell peptide were measured by ELISA after one dose (day 21 pi) or two doses of peptide (day 40 pi). After the first dose, B_2_T-3A induced antibody titers (3.4 ± 0.9 log_10_), whereas no antibodies were detected in any animal from the B_2_T-3D group (Figure 1A). After the peptide boost, antibody titers increased significantly in the B_2_T-3A group (4.1 ± 1.2 log_10_) while only one animal from B_2_T-3D group showed detectable antibodies (Figure 1B).

Next, neutralizing activity against homologous FMDV was analyzed in sera from immunized mice. At day 21 pi, nAbs were detected in animals from B_2_T-3A (1.1 ± 0.6 log_10_). In contrast, none of the mice immunized with B_2_T-3D displayed detectable nAbs (Figure 1C). After the boost, the titers increased in the B_2_T-3A group (2 ± 0.5 log_10_), while only one of the B_2_T-3D immunized mice showed detectable levels of nAbs (Figure 1D).

These results suggest that the T-3D epitope incorporated in the B_2_T-dendrimeric construction is not efficiently recognized as a T-helper epitope in Swiss ICR mice.

### Immunogenicity in Swine of a B_2_T Construction Harboring a T-Cell Epitope From FMDV Non-structural Protein 3D

The above results indicate that the 3D epitope previously identified as a T-cell epitope in swine was not efficiently recognized by murine lymphocytes (Figure 1). To confirm its potential to immunomodulate the response to B_2_T-dendrimers comprising the antigenic B-cell site on the VP1 GH loop in pigs, we decided to test in parallel the immune response elicited by B_2_T-3D and B_2_T-3A in this species, including the longevity of the response. To this end, groups of four pigs were immunized with 2 mg of B_2_T-3A (pigs 80, 81, 82, and 83), B_2_T-3D (pigs 84, 85, 86, and 87) or non-immunized (88 and 89). At day 21 the animals were boosted with the same amount of peptide and sera and PBMCs samples were collected at the indicated times. One animal from B_2_T-3A group (pig 83) showed a deteriorated health status during the second week of the experiment, being excluded from the analysis.

#### Dendrimers B_2_T-3D and B_2_T-3A Elicit Similar Antibody Responses

**Figure 2:**
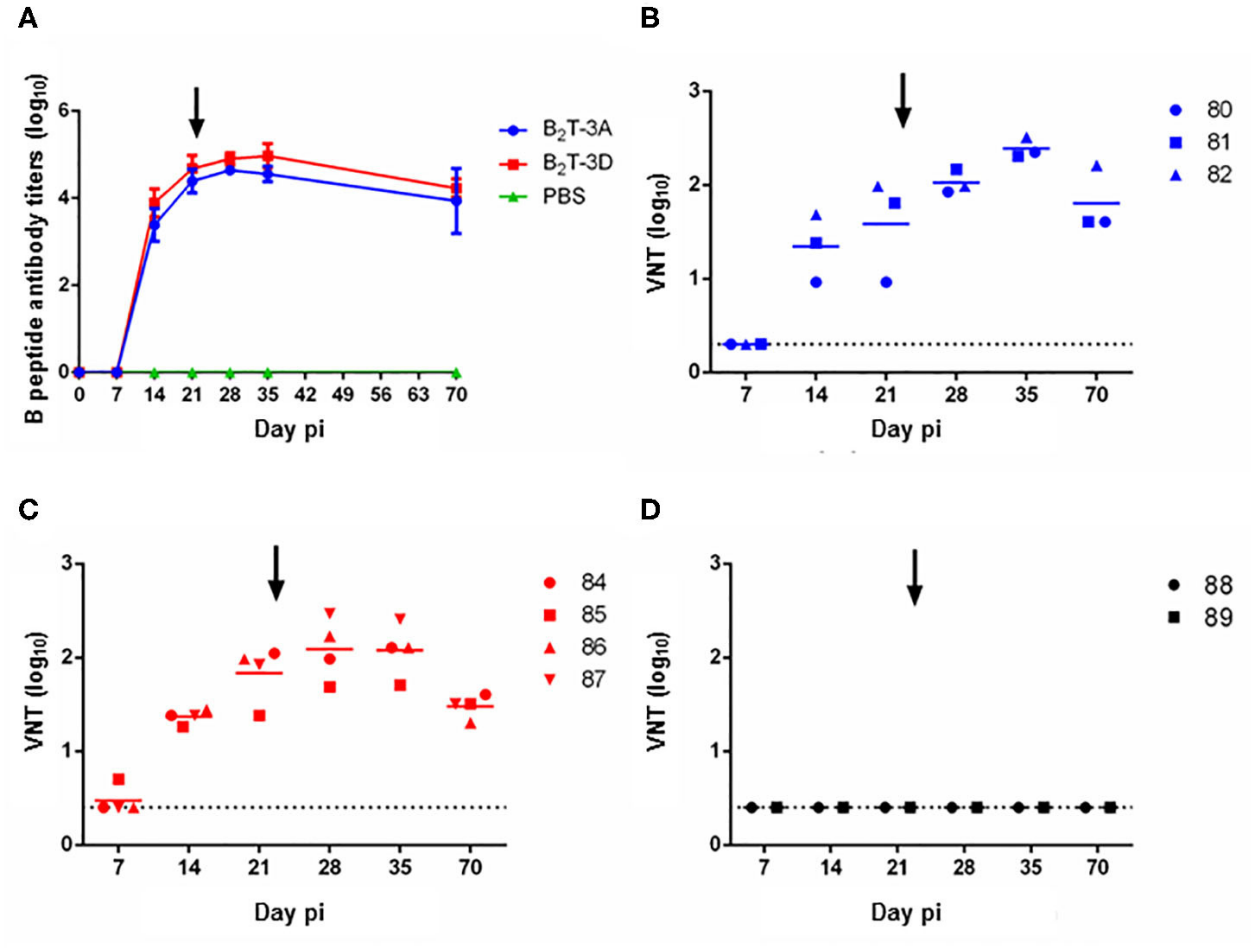
Peptides B_2_T-3D and B_2_T-3A induce similar antibody responses. **(A)** Total IgG specific antibody titers measured by ELISA in sera collected at different days pi. Points depict mean antibody titers for each group of pigs. VNT in sera from animals immunized with **(B)** B_2_T-3A, **(C)** B_2_T-3D, and **(D)** non-immunized. Titers are expressed as the reciprocal log_10_ of the last serum dilution that neutralized 100 TCID50 of homologous FMDV. Each symbol represents the value for an individual pig. Horizontal lines indicate the geometric mean for each animal group (*n* = 4) and dotted lines the detection limit. The arrows show the day of the boost.

The total IgG antibodies elicited by the peptides were measured by ELISA. Specific antibodies were detected in both groups at day 14 pi from which a gradual increment was observed. No remarkable boost effect was observed neither in B_2_T-3A nor in B_2_T-3D immunized pigs and high levels of IgG antibodies were maintained until day 70 (2 months pi) without significant differences between the two groups. As expected, no specific antibodies were detected in the sera from control PBS-inoculated pigs (Figure 2A).

Next, the ability of these antibodies to *in vitro* neutralize homologous virus was tested. B_2_T-3A-immunized pigs elicited nAbs by day 14 pi (1.3 ± 0.4 log_10_) that increased by day 21 pi (1.6 ± 0.6 log_10_). After the second peptide dose, the titers increased reaching an average value of 2 ± 0.1 log_10_ at day 28 pi, and a peak at day 35 pi (2.4 ± 0.1 log_10_), following a gradual smooth decrease until day 70 pi (1.8 ± 0.4 log_10_) (Figure 2B).

The nAbs from B_2_T-3D vaccinated group followed a similar time course and no significant differences were found when compared with B_2_T-3A. At day 14 pi, nAbs titers were first observed (1.4 ± 0.1 log10) and increased at day 21 pi (1.8 ± 0.3 log_10_). After the boost, the average titers reached the peak at day 28 pi (2.1 ± 0.3 log_10_) and were maintained until day 35 pi (2.1 ± 0.3 log_10_). A slight decrease, similar to that observed in the B_2_T-3A group, was detected at day 70 pi (1.5 ± 0.1 log_10_) (Figure 2C). No neutralizing activity was found in sera from PBS-inoculated animals at any time point (Figure 2D).

Thus, B_2_T-3D elicited an antibody response in pigs that paralleled that of B_2_T-3A.

#### T-Cell Responses Elicited by B_2_T-3D and B_2_T-3A

**Figure 3:**
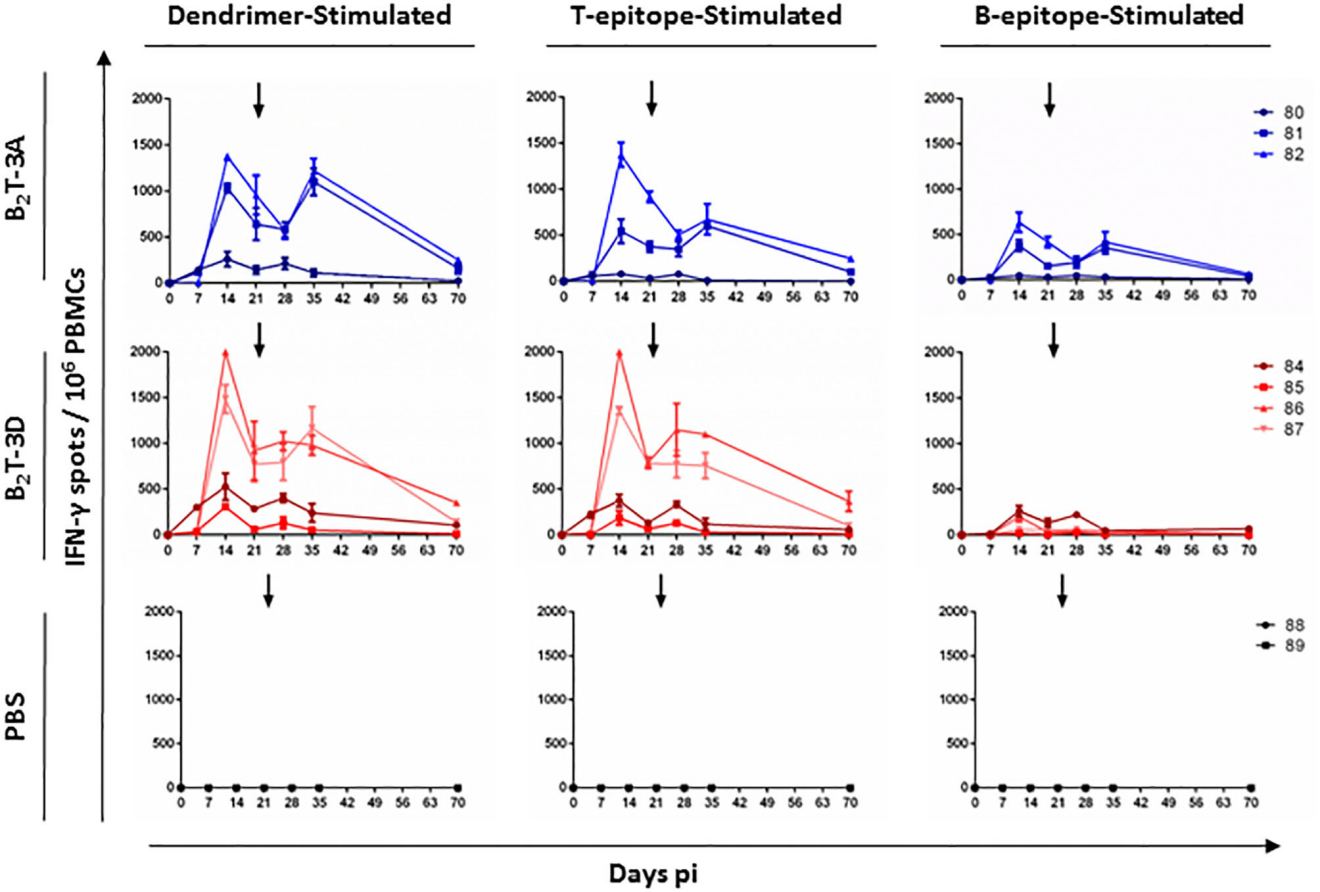
T-cell responses in pigs immunized with B_2_T-3A and B_2_T-3D constructions. PBMCs isolated from individual animals of each group were collected at different days pi. Cells were stimulated *in vitro* for 48 h with homologous dendrimer, T-cell epitope or B-cell epitope, and the number of cells expressing IFN-γ was measured by ELISPOT. PBMCs stimulated with medium (not shown) were included as a negative control of the assay and subtracted. Each point represents the mean of a triplicate of an individual animal. Arrows show the day of the boost.

The ability of peptide B_2_T-3D to induce specific T-cell responses was assessed in PBMCs isolated from immunized pigs by ELISPOT analysis of the IFN-γ-secreting cells. In this experiment, the B-cell peptide was included as stimulus for the *in vitro* recall, to address the possibility of its recognition by T-cells. As in previous experiments, intragroup variability was observed in the responses, which was reflected in the presence in each group of high responders (B_2_T-3A: pigs 81 and 82; B_2_T-3D: pigs 86 and 87) and low responders (B_2_T-3A: pig 80; B_2_T-3D: pigs 84 and 85). A remarkable primary response of IFN-γ secreting cells was noticed at day 14 pi (Figure 3). Interestingly, the two high responder pigs in the B_2_T-3D group showed more IFN-γ spots than the higher responders in the B_2_T-3A group when their PBMCs were stimulated with the whole homologous dendrimer (1.743 ± 364 for B_2_T-3D group vs. 1.206 ± 244 for B_2_T-3A group) and the specific T-cell epitope (1.679 ± 453 vs. 959 ± 587). The magnitude of the responses was lower when cells were stimulated with the B-cell peptide, with the higher values being in pigs immunized with B_2_T-3A (130 ± 97 vs. 507 ± 183) (Figure 3). At day 21 pi the response weaned in both groups reaching similar levels of IFN-γ spots when cells were stimulated with the dendrimer (847 ± 105 vs. 800 ± 224) and the T-cell epitope (785 ± 5 vs. 646 ± 382). At this time, responses against B-cell peptide were clearly lower (42 ± 16 vs. 286 ± 186) (Figure 3). After the boost, a non-immediate secondary response was observed at day 35 pi in the two major responders in each group when stimulated with the corresponding dendrimer (1,073 ± 132 vs. 1,161 ± 87, respectively). However, when stimulated with the T-cell peptide, the IFN-γ production was higher in the B_2_T-3D group (929 ± 242 vs. 637 ± 52). The response dramatically weaned in both groups being scarcely detected at day 70 pi. As expected, non-immunized animals did not induce IFN-γ secreting cells upon stimulation with any of the specific peptides (Figure 3).

Interestingly, the frequencies of IFN-γ spots in response to the B-cell peptide in pigs from the B_2_T-3D group were considerably lower than those of B_2_T-3A immunized animals. These results suggest that the B-cell epitope plays a minor role in cytokine production in B_2_T-3D immunized pigs making T-3D epitope a more potent inducer of IFN-γ-producing cells compared to T-3A epitope.

#### Dendrimers B_2_T-3D and B_2_T-3A Elicit nAbs Against a Broad Spectrum of Type O FMDVs

**Figure 4:**
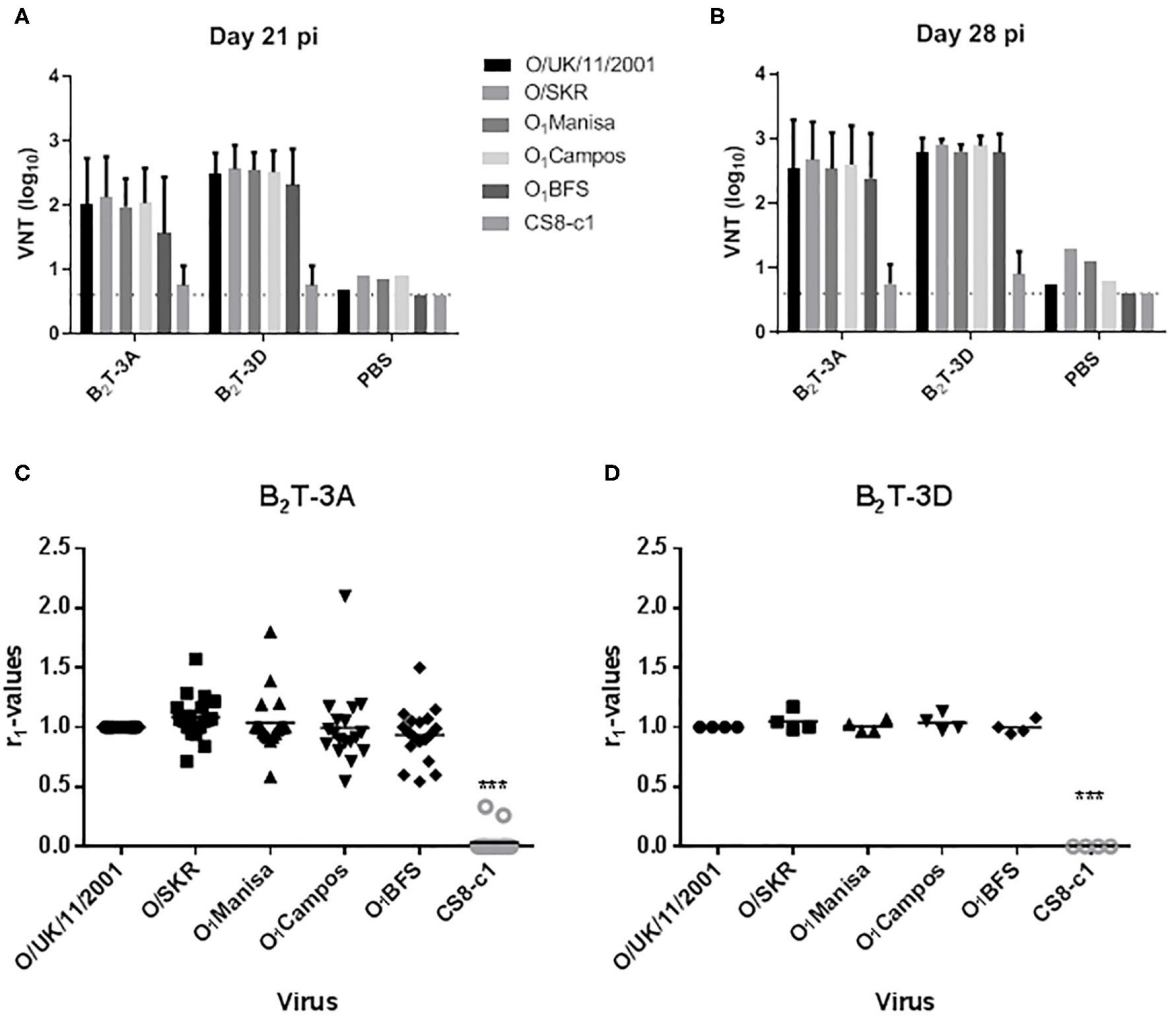
Sera from pigs immunized with B_2_T-dendrimers can neutralize a wide panel of different FMDVs type O topotypes. Sera recovered from animals immunized with B_2_T-3A and B_2_T-3D at days **(A)** 21 and **(B)** 28 pi were tested for its capability to neutralize a panel of different type O FMDVs. Individual columns represent the mean of each group (*n* = 4) ± SD. Values are expressed as the reciprocal log_10_ of the last serum dilution that neutralized 100 TCID50 of each FMDV. **(C,D)** Antigenic relationship (*r*_1_) values of the six viruses. The serological match (r_1_-values; calculated as described in Materials and Methods) of sera from pigs immunized with **(C)** B_2_T-3A including those from previous experiments (20, 31) and **(D)** B_2_T-3D is shown and each symbol represents the value for an individual pig. Horizontal lines indicate the geometric mean for each animal group against each virus (*n* = 18 for B_2_T-3A and *n* = 4 for B_2_T-3D). Statistically significant differences are indicated by asterisks (***) for *p* < 0.0005.

The high antigenic diversity of FMDV makes the development of vaccines a challenging issue. Since type O FMDVs are responsible of many of the current FMD outbreaks in endemic countries, a broad-spectrum response is necessary for optimal vaccines against this serotype (30). Therefore, we were interested in assessing the neutralization range afforded in pigs by the B_2_T-dendrimers studied. To this end, sera recovered from the pigs vaccinated in this study with peptides B_2_T-3A and B_2_T3-D, as well as those of pigs previously immunized with B_2_T-3A (20, 31) were tested for their ability to neutralize a panel of type O FMDVs (Figure 4). The FMDV isolates selected belonged to different type O topotypes, i.e., viruses from different spatiotemporal locations. A non-related serotype C FMDV isolate (CS8-c1) was included as a serotype-specific control.

At day 21 pi, animals immunized with B_2_T-3A and B_2_T-3D showed nAbs titers against the panel of FMDVs without significant differences among the viruses compared (Figure 4A). A similar neutralizing profile was observed at day 28 pi, after the second immunization with the same dose of each of the peptides (Figure 4B). The level of neutralization afforded at day 21 pi by B_2_T-3A and B_2_T-3D against the panel of viruses relative to the homologous isolate O/UK/11/2001 (r1 value; VNT ratio: virus problem/O/UK/11/2001) is shown in Figures 4C,D. While for B_2_T-3D the data compared belong to the four pigs immunized in this study (Figure 4D), for B_2_T-3A, a total of 18 animals, including those immunized with this dendrimer in previous works (20, 31) were included (Figure 4C). All the viruses tested showed r1 values similar or for some of the animals immunized with B_2_T-3A even higher than that of O/UK/11/2001. As expected, the nAbs were serotype-specific and none of the sera from any immunized animal was able to neutralize type C CS8-c1 virus (Figure 4).

These results support that B_2_T-dendrimers induce a broad anti-FMDV immunity within a serotype, which can be considered as an important valuable asset for their potential use in endemic countries where a wide spectrum of antigenic variable pools of FMDVs can circulate.

## Discussion

For a rational FMDV peptide vaccine design, the incorporation of B-cell antigenic sites that fully mimic their native viral conformation and are efficiently recognized by B-cells, as well as species embodying T-cell epitopes that provide an adequate T-cell help and recognition from lymphocytes, are key issues. In this context, the characterization of FMDV-specific epitopes functionally analogous to T-3A is a relevant work to extend the repertoire of T-cell epitopes to be included in dendrimeric vaccines that particularly face the MHC (SLA in swine) polymorphisms of different pig breeding's, thus becoming a potential manner to increase the quality of the immune responses elicited by peptide-based vaccines.

We previously reported that alternative subunit vaccines consisting of multiple FMDV antigenic peptides, including B_2_T-3A, induced similar nAb titers in outbred Swiss mice as those elicited in pigs (31, 32). Therefore, in this work we first addressed the immunogenicity of B_2_T-3D dendrimer peptide, using this mouse model as a screening system to confirm that this construction, harboring a T-cell epitope identified in swine, retained the ability to elicit FMDV nAbs. Unexpectedly, our results indicate that peptide B_2_T-3D did not induce Ab in mice unlike the previous analog B_2_T-3A. These results suggest that the T-3D epitope comprised in the B_2_T-dendrimeric platform is not efficiently recognized as a T-helper epitope in Swiss mice, probably due to its low affinity for mouse MHC class II haplotypes. Nevertheless, we cannot rule out that an inefficient processing of the epitope and/or conformation alterations affecting to the correct cross-linking of the B-epitope can also be contributing to the lack of antibody induction observed. Thus, further work is required to confirm the lack of recognition of T-3D by murine T-cells. In any case, our results evidence the limitations of mouse models for the analysis of the role of FMDV-specific T-cell epitopes (33–35).

Replacement of T-3A or its combination with other T-cell peptides are possibilities to explore the effect of altering the recognition B_2_T constructions by T-cells. As commented above, different T-cell epitopes previously identified in swine were not efficiently recognized by murine lymphocytes. Thus, despite the limited amount of nAbs elicited by B_2_T-3D in mice, we selected T-3D to study the effect of its inclusion on the immunogenicity of B_2_T-dendrimer in swine, showing that B_2_T-3D and B_2_T-3A elicited similar antibody responses, with titers being consistent by day 70 pi, as previously reported for B_2_T-3A (31).

Animal-to-animal variation observed is a common feature in previous studies with peptide and other subunit vaccines (9, 11, 18, 31, 36, 37). As in all FMDV natural hosts, the genetic background of individual pigs may differ. Thus, a pool of SLA alleles exists in the population and, as mentioned above, this polymorphism can contribute to the individual variability observed (38).

Both B_2_T-3A and B_2_T-3D elicited consistent levels of neutralizing antibodies. As previously reported, B_2_T-3A also induced IFN-γ expressing T-cells that were *in vitro* recalled by T-3A peptide and, interestingly to a lower extent, by B-cell peptide with similar time courses, supporting that both sequences were recognized as T-cell epitopes. Conversely, the IFN-γ expressing cells elicited by B_2_T-3D preferentially recognized the T-3D peptide, suggesting that this epitope is a potent inducer of IFN-γ. Further experiments are in progress to confirm the immunostimulatory differences between T-3A and T-3D.

Implementation of efficient vaccination campaigns against FMD requires the use of inactivated viruses capable of eliciting protective responses against circulating and emerging FMDVs, including serotype- specific vaccine isolates into vaccine formulations (39). Thus, because of the wide antigenic range presented by FMDV, an optimal vaccine needs to protect against a wide FMDV spectrum. This is particularly the case for vaccines against type O viruses, which are responsible for major outbreaks in epidemic countries (40).

Initial experiments with linear peptides indicated that the elicited nAbs were able to neutralize not only the homologous virus, whose sequence contains the VP1 GH-loop, but also heterologous FMDV isolates (41). Our results show that dendrimer peptide B_2_T-3D elicited, in most cases, high titers of cross-neutralizing antibodies, which, for some isolates, were higher than those against the homologous virus in a similar manner than when using B_2_T-3A to immunize pigs. Multiple factors inherent to the assay such as the differences in thermal stability among the viral isolates analyzed, can contribute to explain these observations, which have also been reported for type A FMDV conventionally vaccinated animals (27) and for an adenovirus-vectored type O FMDV vaccine (42). On the other hand, the modular approach used also allows extension to other FMDV serotypes.

In summary, a B_2_T-dendrimer incorporating FMDV T-cell epitope T-3D elicits high levels of neutralizing antibodies and a potent response of IFN-γ producing-cells. These results extend the repertoire of T-cell epitopes efficiently recognized by swine lymphocytes and open the possibility of using T-3D to enhance the immunogenicity and the protection conferred by B_2_T-dendrimers.

## Data Availability Statement

All datasets presented in this study are included in the article/supplementary material.

## Ethics Statement

The animal study was reviewed and approved by CSIC Committees on Ethical and Animal Welfare and by the National Committee on Ethics and Animal Welfare (PROEX 034/15) (CBS2014/015 and CEEA2014/018) by the INIA Committees on Ethics of Animal Experiments and Biosafety, and by the National Committee on Ethics and Animal Welfare (PROEX 218/14).

## Author Contributions

FS, EB, DA, RC-A, PL, and MF conceived and designed the experiments. RC-A, PL, ET, MF, SD, and MB performed the experiments. RC-A, PL, MF, EB, DA, and FS analyzed the data and wrote the manuscript. All authors contributed to manuscript revision, read, and approved the submitted version.

## Conflict of Interest

The authors declare that the research was conducted in the absence of any commercial or financial relationships that could be construed as a potential conflict of interest.

## References

[1] Knight-Jones TJ Robinson L Charleston B Rodriguez LL Gay CG Sumption KJ. Global foot-and-mouth disease research update and gap analysis: 1 - overview of global status and research needs. Transbound Emerg Dis. (2016) 63 (Suppl. 1):3–13. 10.1111/tbed.12528 27320162

[2] Grubman MJ Baxt B. Foot-and-mouth disease. Clin Microbiol Rev. (2004) 17:465–93. 10.1128/CMR.17.2.465-493.2004 15084510 PMC387408

[3] Cao Y Lu Z Liu Z. Foot-and-mouth disease vaccines: progress and problems. Expert Rev Vaccines. (2016) 15:783–9. 10.1586/14760584.2016.1140042 26760264

[4] Parida S. Vaccination against foot-and-mouth disease virus: strategies and effectiveness. Expert Rev Vaccines. (2009) 8:347–65. 10.1586/14760584.8.3.347 19249976

[5] Robinson L Knight-Jones TJ Charleston B Rodriguez LL Gay CG Sumption KJ Global foot-and-mouth disease research update and gap analysis: 3 - vaccines. Transbound Emerg Dis. (2016) 63 (Suppl. 1):30–41. 10.1111/tbed.12521 27320164

[6] Kleid DG Yansura D Small B Dowbenko D Moore DM Grubman MJ. Cloned viral protein vaccine for foot-and-mouth disease: responses in cattle and swine. Science. (1981) 214:1125–9. 10.1126/science.6272395 6272395

[7] Bittle JL Houghten RA Alexander H Shinnick TM Sutcliffe JG Lerner RA. Protection against foot-and-mouth disease by immunization with a chemically synthesized peptide predicted from the viral nucleotide sequence. Nature. (1982) 298:30–3. 10.1038/298030a0 7045684

[8] DiMarchi R Brooke G Gale C Cracknell V Doel T Mowat N. Protection of cattle against foot-and-mouth disease by a synthetic peptide. Science. (1986) 232:639–41. 10.1126/science.3008333 3008333

[9] Wang CY Chang TY Walfield AM Ye J Shen M Chen SP. Effective synthetic peptide vaccine for foot-and-mouth disease in swine. Vaccine. (2002) 20:2603–10. 10.1016/S0264-410X(02)00148-2 12057619

[10] Doel TR. Natural and vaccine induced immunity to FMD. Curr Top Microbiol Immunol. (2005) 288:103–31. 10.1007/3-540-27109-0_5 15648176

[11] Taboga O Tami C Carrillo E Nunez JI Rodriguez A Saiz JC. A large-scale evaluation of peptide vaccines against foot-and-mouth disease: lack of solid protection in cattle and isolation of escape mutants. J Virol. (1997) 71:2606–14. 10.1128/JVI.71.4.2606-2614.1997 9060612 PMC191381

[12] Collen T. Foot-and-mouth disease virus (aphthovirus): viral T cell epitopes. In: Goddeevis BML, Morrison I, editor. Cell Mediated Immunity in Ruminants. Boca Raton: CRC Press Inc. (1994). p. 173–97.

[13] Cubillos C de la Torre BG Barcena J Andreu D Sobrino F Blanco E. Inclusion of a specific T cell epitope increases the protection conferred against foot-and-mouth disease virus in pigs by a linear peptide containing an immunodominant B cell site. Virol J. (2012) 9:66. 10.1186/1743-422X-9-66 22416886 PMC3313860

[14] Glass EJ Oliver RA Collen T Doel TR Dimarchi R Spooner RL. MHC class II restricted recognition of FMDV peptides by bovine T cells. Immunology. (1991) 74:594–9. 1664415 PMC1384766

[15] Sobrino F Blanco E Garcia-Briones M Ley V. Synthetic peptide vaccines: foot-and-mouth disease virus as a model. Dev Biol Stand. (1999) 101:39–43. 10566773

[16] Rodriguez LL Barrera J Kramer E Lubroth J Brown F Golde WT A synthetic peptide containing the consensus sequence of the G-H loop region of foot-and-mouth disease virus type-O VP1 and a promiscuous T-helper epitope induces peptide-specific antibodies but fails to protect cattle against viral challenge. Vaccine. (2003) 21:3751–6. 10.1016/S0264-410X(03)00364-5 12922108

[17] Tam JP. Synthetic peptide vaccine design: synthesis and properties of a high-density multiple antigenic peptide system. Proc Natl Acad Sci USA. (1988) 85:5409–13. 10.1073/pnas.85.15.5409 3399498 PMC281766

[18] Cubillos C de la Torre BG Jakab A Clementi G Borras E Barcena J. Enhanced mucosal immunoglobulin A response and solid protection against foot-and-mouth disease virus challenge induced by a novel dendrimeric peptide. J Virol. (2008) 82:7223–30. 10.1128/JVI.00401-08 18448530 PMC2446959

[19] Blanco E Guerra B de la Torre BG Defaus S Dekker A Andreu D. Full protection of swine against foot-and-mouth disease by a bivalent B-cell epitope dendrimer peptide. Antiviral Res. (2016) 129:74–80. 10.1016/j.antiviral.2016.03.005 26956030

[20] Canas-Arranz R Forner M Defaus S de Leon P Bustos MJ Torres E. A single dose of dendrimer B2T peptide vaccine partially protects pigs against foot-and-mouth disease virus infection. Vaccines (Basel). (2020) 8:19. 10.3390/vaccines8010019 31936706 PMC7157199

[21] McCullough KC Sobrino F Immunology of Foot-and-mouth disease. In: Sobrino F. Domingo E, editor. Foot and Mouth Disease: Current Perspectives. Norfolk: Horizon Bioscience (2004) 173–222. 10.1201/9781420037968.ch8

[22] Collen T Doel TR. Heterotypic recognition of foot-and-mouth disease virus by cattle lymphocytes. J Gen Virol. (1990) 71 (Pt 2):309–15. 10.1099/0022-1317-71-2-309 1689767

[23] Garcia-Briones MM Russell GC Oliver RA Tami C Taboga O Carrillo E. Association of bovine DRB3 alleles with immune response to FMDV peptides and protection against viral challenge. Vaccine. (2000) 19:1167–71. 10.1016/S0264-410X(00)00313-3 11137253

[24] Garcia-Briones MM Blanco E Chiva C Andreu D Ley V Sobrino F. Immunogenicity and T cell recognition in swine of foot-and-mouth disease virus polymerase 3D. Virology. (2004) 322:264–75. 10.1016/j.virol.2004.01.027 15110524

[25] Monso M de la Torre BG Blanco E Moreno N Andreu D. Influence of conjugation chemistry and B epitope orientation on the immune response of branched peptide antigens. Bioconjug Chem. (2013) 24:578–85. 10.1021/bc300515t 23458489

[26] Sobrino F Davila M Ortin J Domingo E. Multiple genetic variants arise in the course of replication of foot-and-mouth disease virus in cell culture. Virology. (1983) 128:310–8. 10.1016/0042-6822(83)90258-1 6310859

[27] Bari FD Parida S Tekleghiorghis T Dekker A Sangula A Reeve R. Genetic and antigenic characterisation of serotype A FMD viruses from East Africa to select new vaccine strains. Vaccine. (2014) 32:5794–800. 10.1016/j.vaccine.2014.08.033 25171846 PMC4194315

[28] Borrego B Blanco E Rodriguez Pulido M Mateos F Lorenzo G Cardillo S. Combined administration of synthetic RNA and a conventional vaccine improves immune responses and protection against foot-and-mouth disease virus in swine. Antiviral Res. (2017) 142:30–6. 10.1016/j.antiviral.2017.03.009 28315707

[29] Habiela M Seago J Perez-Martin E Waters R Windsor M Salguero FJ. Laboratory animal models to study foot-and-mouth disease: a review with emphasis on natural and vaccine-induced immunity. J Gen Virol. (2014) 95:2329–45. 10.1099/vir.0.068270-0 25000962 PMC4202264

[30] Mahapatra M Parida S. Foot and mouth disease vaccine strain selection: current approaches and future perspectives. Expert Rev Vaccines. (2018) 17:577–91. 10.1080/14760584.2018.1492378 29950121

[31] Canas-Arranz R Forner M Defaus S Rodriguez-Pulido M de Leon P Torres E. A bivalent B-cell epitope dendrimer peptide can confer long-lasting immunity in swine against foot-and-mouth disease. Transbound Emerg Dis. (2020b). 10.1111/tbed.13497 31994334

[32] Blanco E Cubillos C Moreno N Barcena J de la Torre BG Andreu D. B epitope multiplicity and B/T epitope orientation influence immunogenicity of foot-and-mouth disease peptide vaccines. Clin Dev Immunol. (2013) 2013:475960. 10.1155/2013/475960 24454475 PMC3878600

[33] Cunliffe HR Blackwell JH. Survival of foot-and-mouth disease virus in casein and sodium caseinate produced from the milk of infected cows (1). J Food Prot. (1977) 40:389–92. 10.4315/0362-028X-40.6.389 30731643

[34] Langellotti C Quattrocchi V Alvarez C Ostrowski M Gnazzo V Zamorano P. Foot-and-mouth disease virus causes a decrease in spleen dendritic cells and the early release of IFN-alpha in the plasma of mice. Differences between infectious and inactivated virus. Antiviral Res. (2012) 94:62–71. 10.1016/j.antiviral.2012.02.009 22387627

[35] McVicar JW Richmond JY Campbell CH Hamilton LD. Observations of cattle, goats and pigs after administration of synthetic interferon inducers and subsequent exposure to foot and mouth disease virus. Can J Comp Med. (1973) 37:362–8. 4356316 PMC1319793

[36] Gullberg M Lohse L Botner A McInerney GM Burman A Jackson T. A prime-boost vaccination strategy in cattle to prevent foot-and-mouth disease using a “single-cycle” alphavirus vector and empty capsid particles. PLoS ONE. (2016) 11:e0157435. 10.1371/journal.pone.0157435 27294397 PMC4905628

[37] Soria I Quattrocchi V Langellotti C Gammella M Digiacomo S Garcia de la Torre B. Dendrimeric peptides can confer protection against foot-and-mouth disease virus in cattle. PLoS ONE. (2017) 12:e0185184. 10.1371/journal.pone.0185184 28949998 PMC5614567

[38] Hammer SE Ho CS Ando A Rogel-Gaillard C Charles M Tector M. Importance of the major histocompatibility complex (swine leukocyte antigen) in swine health and biomedical research. Annu Rev Anim Biosci. (2020) 8:171–98. 10.1146/annurev-animal-020518-115014 31846353

[39] Doel TR. FMD vaccines. Virus Res. (2003) 91:81–99. 10.1016/S0168-1702(02)00261-7 12527439

[40] Knowles NJ Samuel AR. Molecular epidemiology of foot-and-mouth disease virus. Virus Res. (2003) 91:65–80. 10.1016/S0168-1702(02)00260-5 12527438

[41] Parry NR Ouldridge EJ Barnett PV Clarke BE Francis MJ Fox JD. Serological prospects for peptide vaccines against foot-and-mouth disease virus. J Gen Virol. (1989) 70:2919–30. 10.1099/0022-1317-70-11-2919 2479714

[42] Fernandez-Sainz I Medina GN Ramirez-Medina E Koster MJ Grubman MJ de Los Santos T. Adenovirus-vectored foot-and-mouth disease vaccine confers early and full protection against FMDV O1 Manisa in swine. Virology. (2017) 502:123–32. 10.1016/j.virol.2016.12.021 28039799

